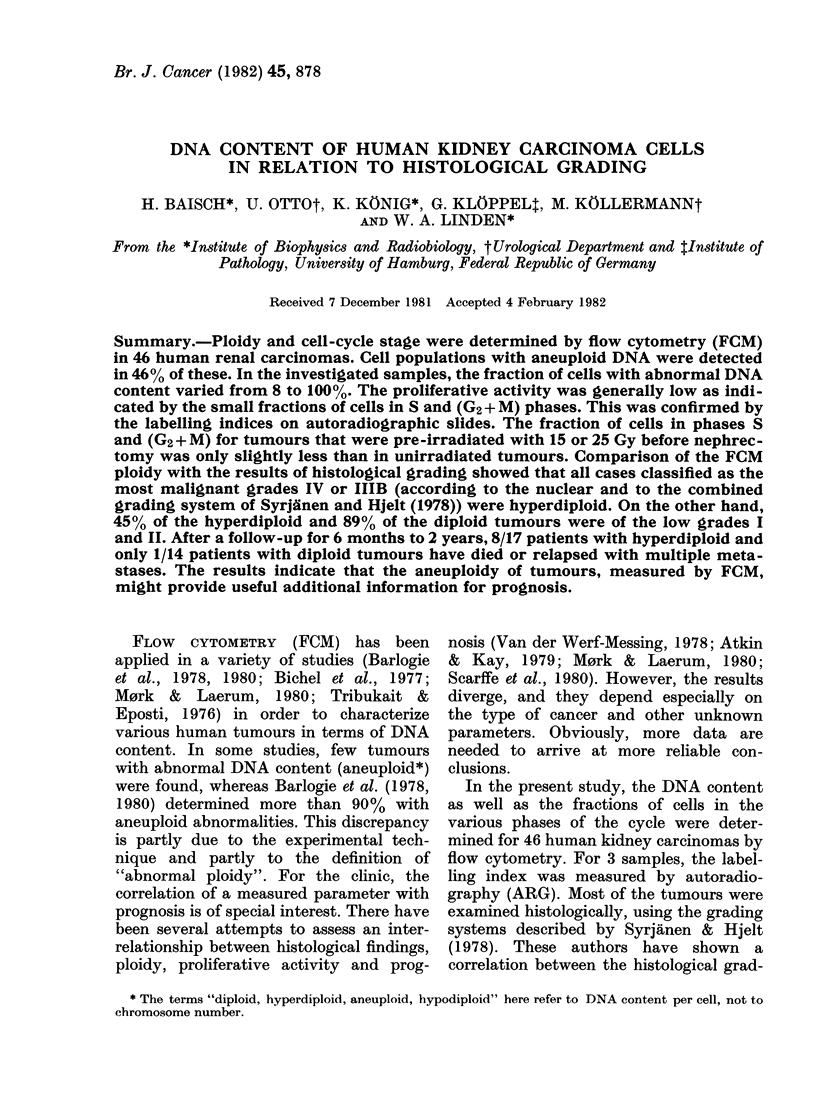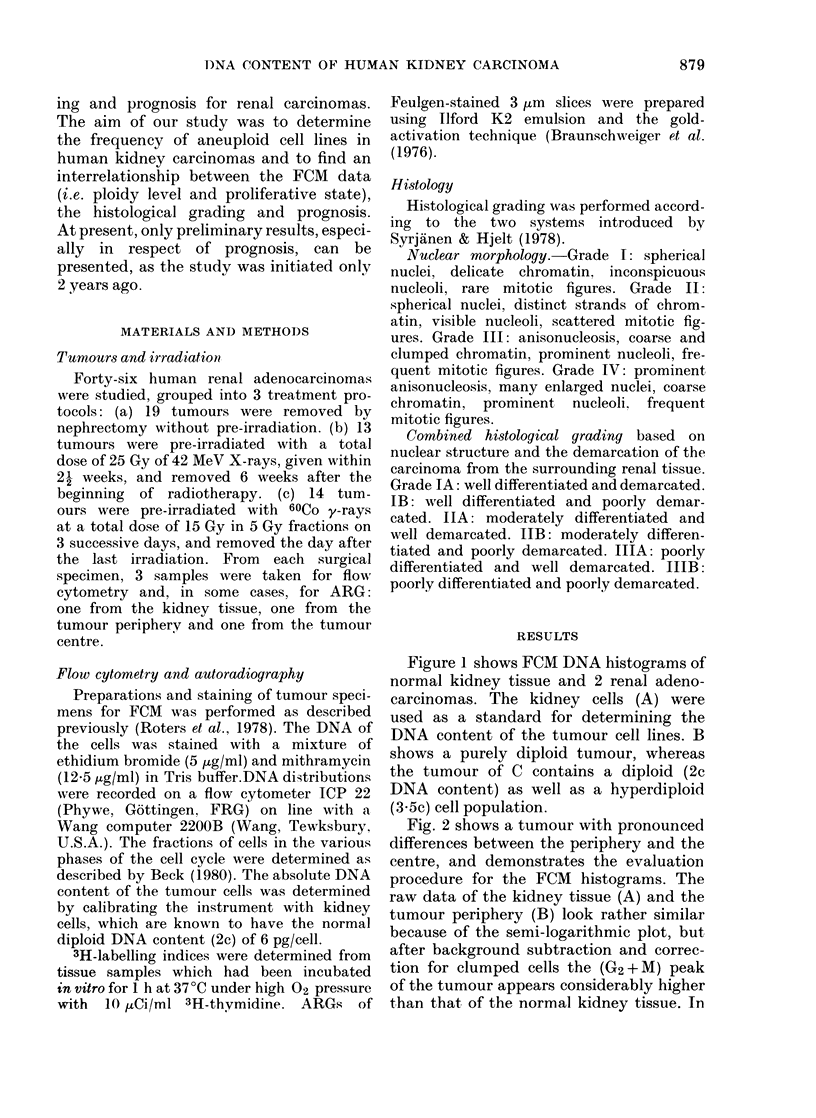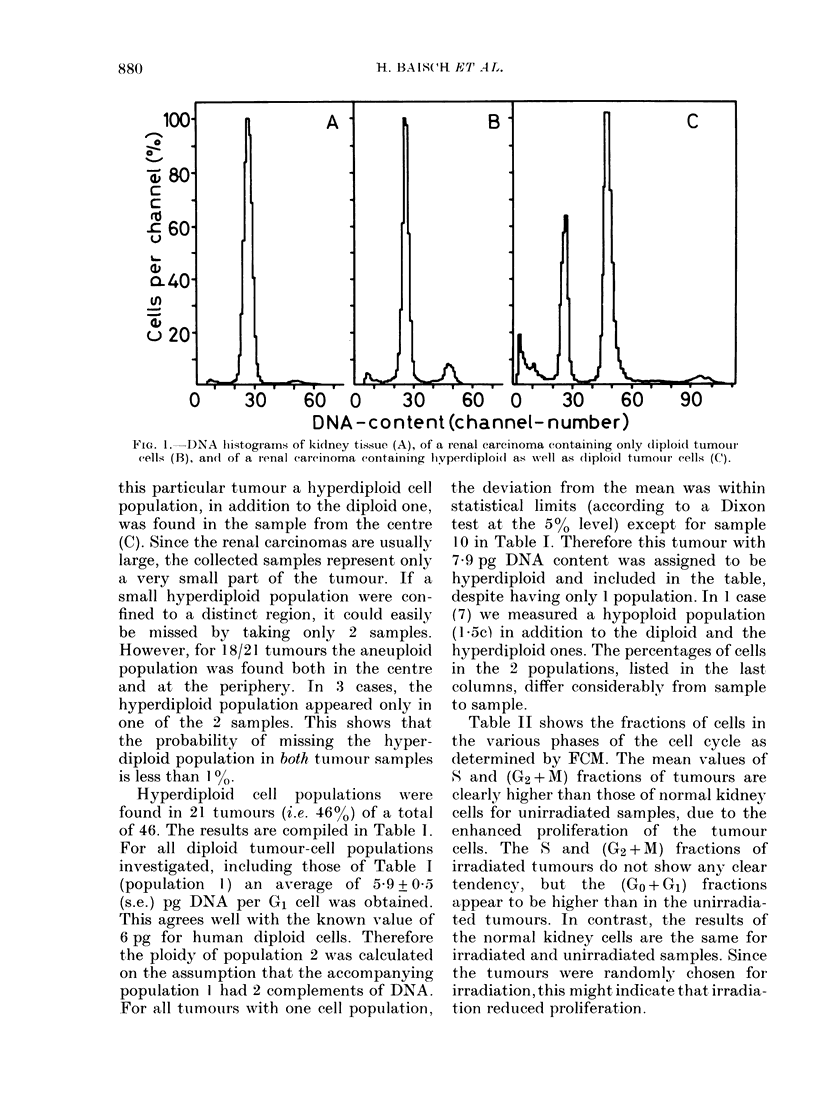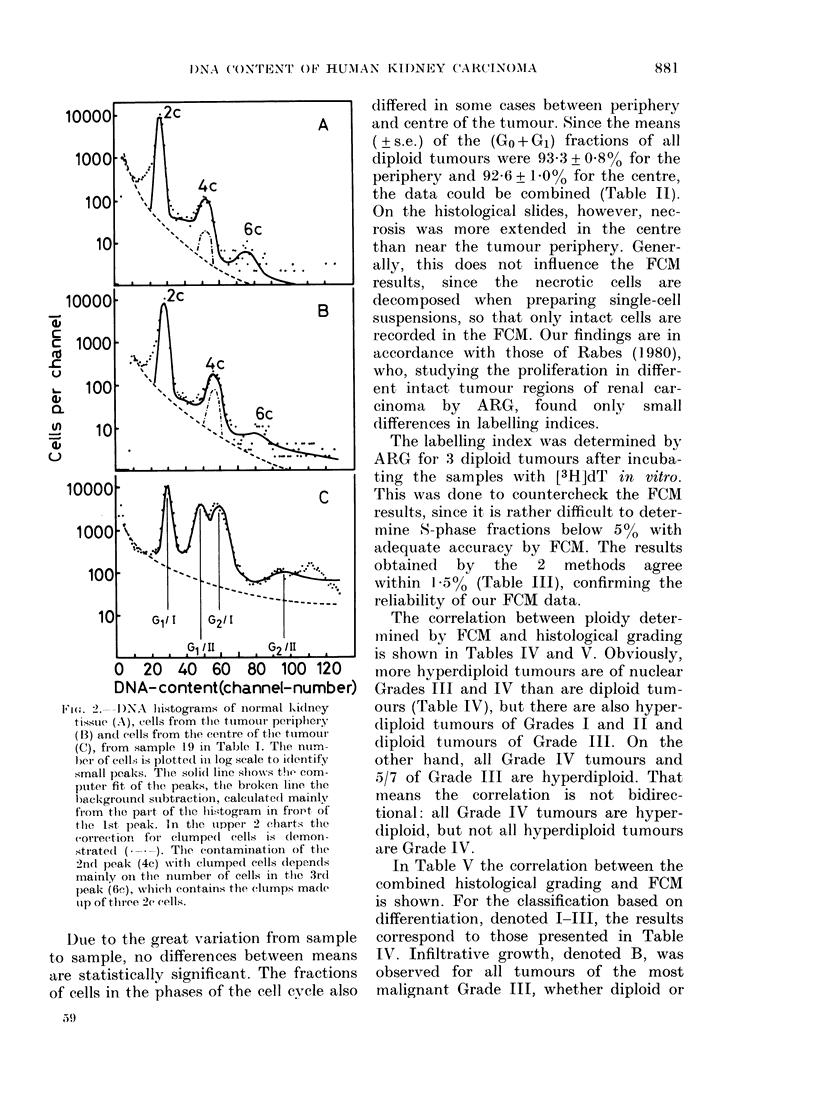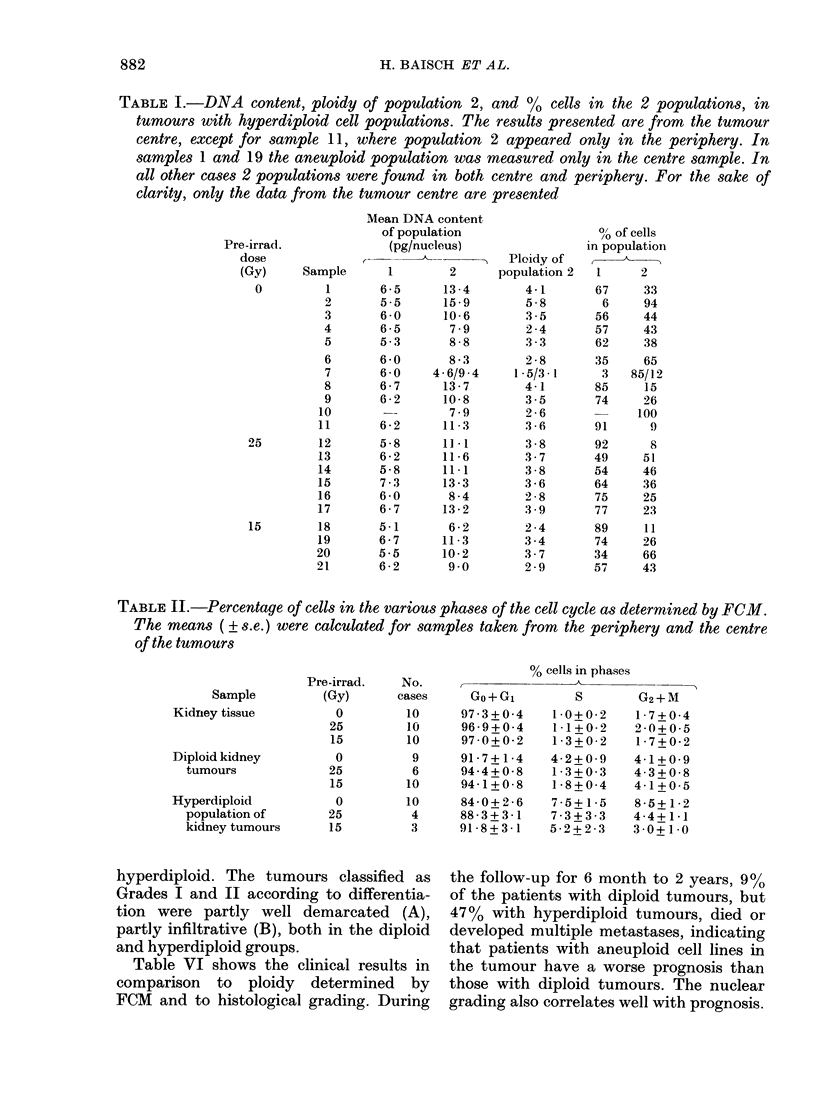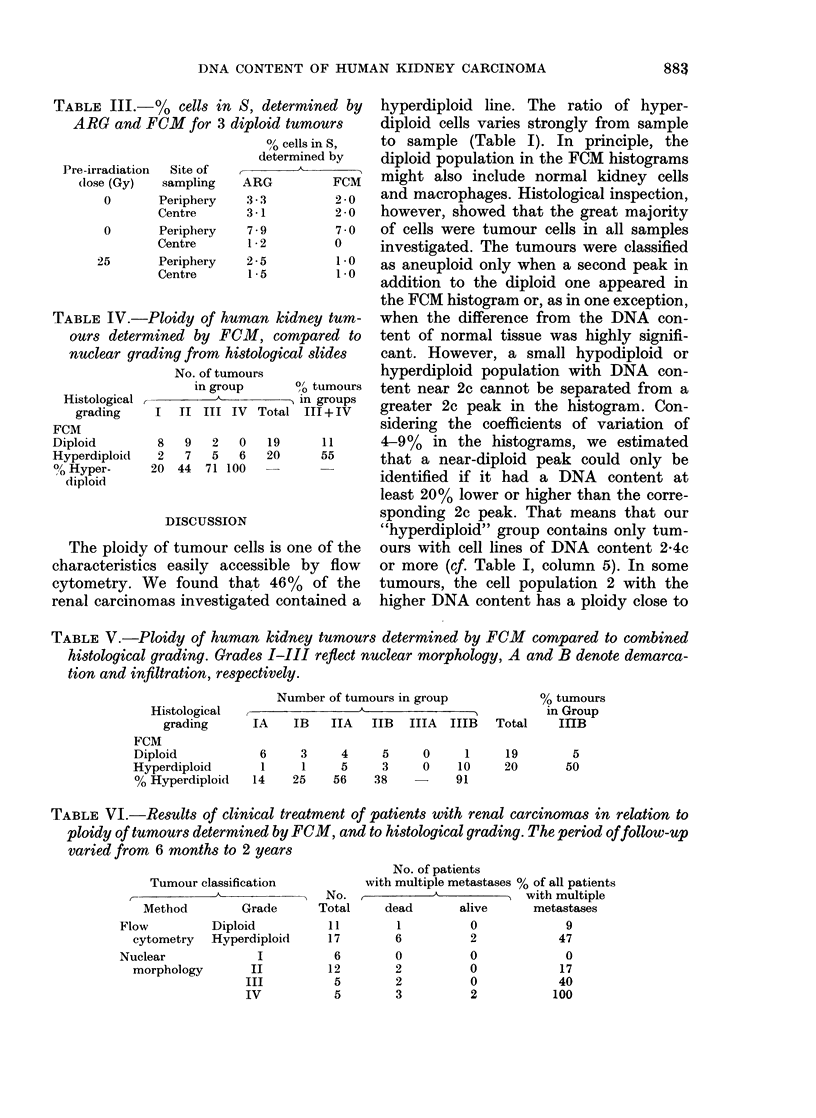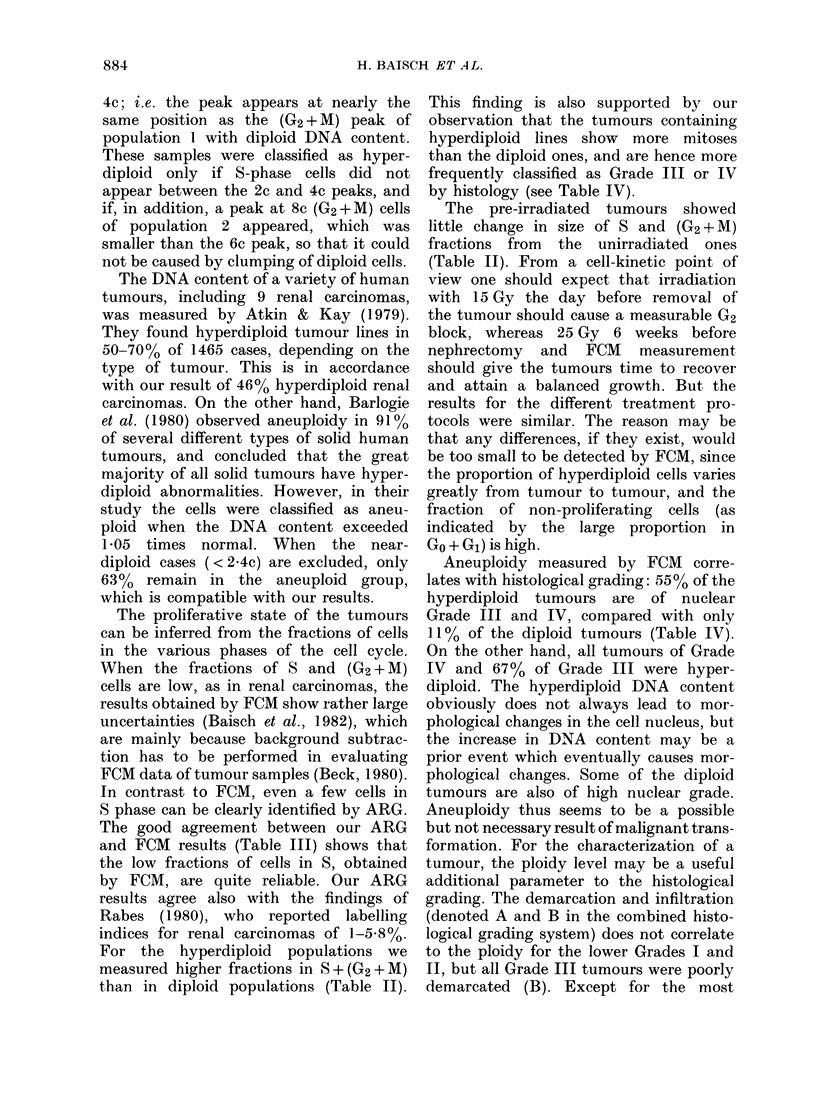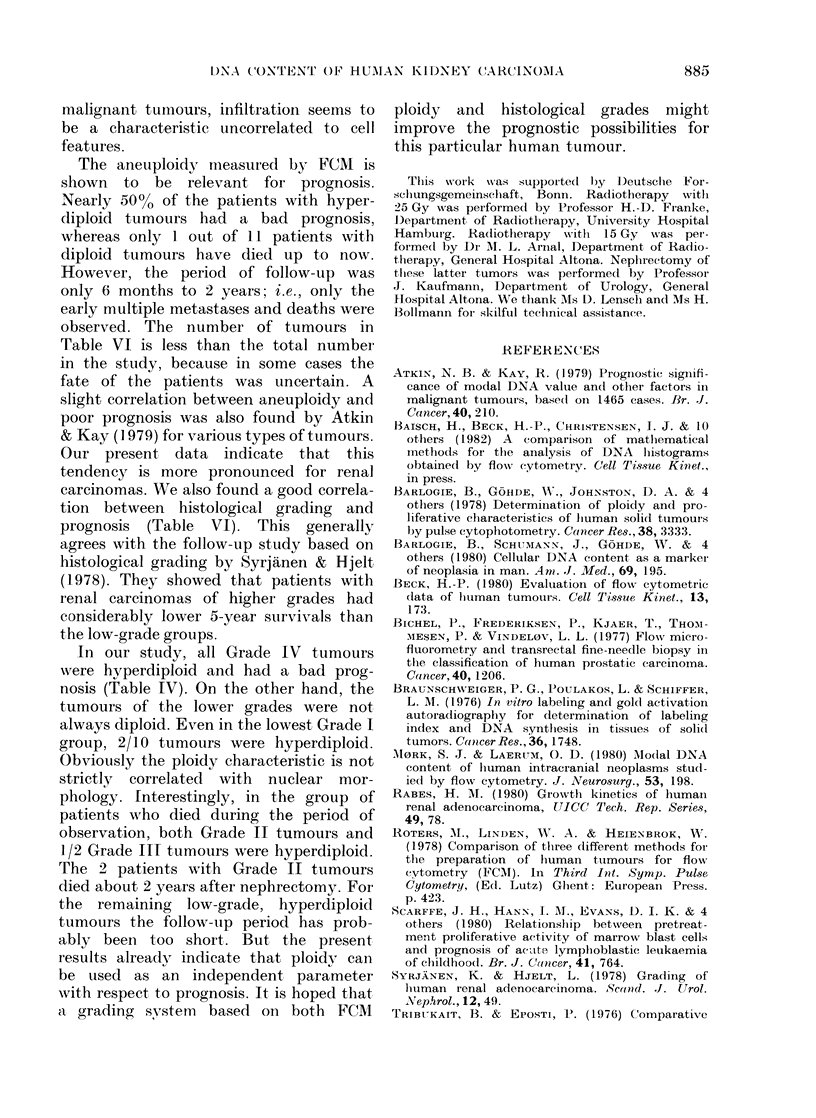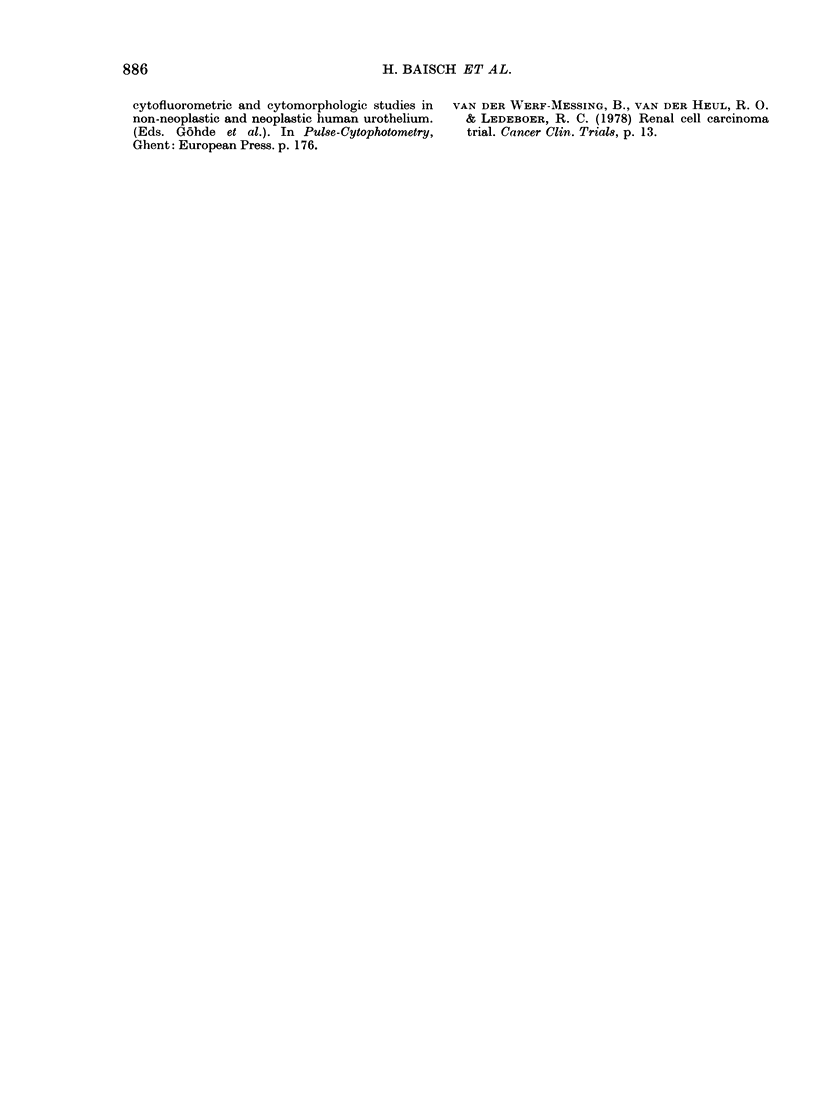# DNA content of human kidney carcinoma cells in relation to histological grading.

**DOI:** 10.1038/bjc.1982.140

**Published:** 1982-06

**Authors:** H. Baisch, U. Otto, K. König, G. Klöppel, M. Köllermann, W. A. Linden

## Abstract

Ploidy and cell-cycle stage were determined by flow cytometry (FCM) in 46 human renal carcinomas. Cell populations with aneuploid DNA were detected in 46% of these. In the investigated samples, the fraction of cells with abnormal DNA content varied from 8 to 100%. The proliferative activity was generally low as indicated by the small fractions of cells in S and (G2 + M) phases. This was confirmed by the labelling indices on autoradiographic slides. The fraction of cells in phases S and (G2 + M) for tumours that were pre-irradiated with 15 or 25 Gy before nephrectomy was only slightly less than in unirradiated tumours. Comparison of the FCM ploidy with the results of histological grading showed that all cases classified as the most malignant grades IV or IIIB (according to the nuclear and to the combined grading system of Syrjänen and Hjelt (1978) were hyperdiploid. On the other hand, 45% of the hyperdiploid and 89% of the diploid tumours were of the low grades I and II. After a follow-up for 6 months to 2 years, 8/17 patients with hyperdiploid and only 1/14 patients with diploid tumours have died or relapsed with multiple metastases. The results indicate that the aneuploidy of tumours, measured by FCM, might provide useful additional information for prognosis.


*Br. J. Cancer* (1982) **45**, 878

### DNA CONTENT OF HUMAN KIDNEY CARCINOMA CELLS IN RELATION TO HISTOLOGICAL GRADING

H. BAISCH*, U. OTTO†, K. KÖNIG*, G. KLÖPPEL‡, M. KÖLLERMANN† AND W. A. LINDEN*

*From the *Institute of Biophysics and Radiobiology, †Urological Department and ‡Institute of Pathology, University of Hamburg, Federal Republic of Germany*

Received 7 December 1981 Accepted 4 February 1982

**Summary.—Ploidy and cell-cycle stage were determined by flow cytometry (FCM) in 46 human renal carcinomas. Cell populations with aneuploid DNA were detected in 46% of these. In the investigated samples, the fraction of cells with abnormal DNA content varied from 8 to 100%. The proliferative activity was generally low as indicated by the small fractions of cells in S and ($G_2+M$) phases. This was confirmed by the labelling indices on autoradiographic slides. The fraction of cells in phases S and ($G_2+M$) for tumours that were pre-irradiated with 15 or 25 Gy before nephrectomy was only slightly less than in unirradiated tumours. Comparison of the FCM ploidy with the results of histological grading showed that all cases classified as the most malignant grades IV or IIIB (according to the nuclear and to the combined grading system of Syrjänen and Hjelt (1978)) were hyperdiploid. On the other hand, 45% of the hyperdiploid and 89% of the diploid tumours were of the low grades I and II. After a follow-up for 6 months to 2 years, 8/17 patients with hyperdiploid and only 1/14 patients with diploid tumours have died or relapsed with multiple metastases. The results indicate that the aneuploidy of tumours, measured by FCM, might provide useful additional information for prognosis.**

FLOW CYTOMETRY (FCM) has been applied in a variety of studies (Barlogie *et al.*, 1978, 1980; Bichel *et al.*, 1977; Mørk & Laerum, 1980; Tribukait & Eposti, 1976) in order to characterize various human tumours in terms of DNA content. In some studies, few tumours with abnormal DNA content (aneuploid*) were found, whereas Barlogie *et al.* (1978, 1980) determined more than 90% with aneuploid abnormalities. This discrepancy is partly due to the experimental technique and partly to the definition of "abnormal ploidy". For the clinic, the correlation of a measured parameter with prognosis is of special interest. There have been several attempts to assess an interrelationship between histological findings, ploidy, proliferative activity and prognosis (Van der Werf-Messing, 1978; Atkin & Kay, 1979; Mørk & Laerum, 1980; Scarffe *et al.*, 1980). However, the results diverge, and they depend especially on the type of cancer and other unknown parameters. Obviously, more data are needed to arrive at more reliable conclusions.

In the present study, the DNA content as well as the fractions of cells in the various phases of the cycle were determined for 46 human kidney carcinomas by flow cytometry. For 3 samples, the labelling index was measured by autoradiography (ARG). Most of the tumours were examined histologically, using the grading systems described by Syrjänen & Hjelt (1978). These authors have shown a correlation between the histological grad-

* The terms "diploid, hyperdiploid, aneuploid, hypodiploid" here refer to DNA content per cell, not to chromosome number.

ing and prognosis for renal carcinomas. The aim of our study was to determine the frequency of aneuploid cell lines in human kidney carcinomas and to find an interrelationship between the FCM data (*i.e.* ploidy level and proliferative state), the histological grading and prognosis. At present, only preliminary results, especially in respect of prognosis, can be presented, as the study was initiated only 2 years ago.

### MATERIALS AND METHODS

### *Tumours and irradiation*

Forty-six human renal adenocarcinomas were studied, grouped into 3 treatment protocols: (a) 19 tumours were removed by nephrectomy without pre-irradiation. (b) 13 tumours were pre-irradiated with a total dose of 25 Gy of 42 MeV X-rays, given within $2\frac{1}{2}$ weeks, and removed 6 weeks after the beginning of radiotherapy. (c) 14 tumours were pre-irradiated with $^{60}$Co $\gamma$-rays at a total dose of 15 Gy in 5 Gy fractions on 3 successive days, and removed the day after the last irradiation. From each surgical specimen, 3 samples were taken for flow cytometry and, in some cases, for ARG: one from the kidney tissue, one from the tumour periphery and one from the tumour centre.

### *Flow cytometry and autoradiography*

Preparations and staining of tumour specimens for FCM was performed as described previously (Roters *et al.*, 1978). The DNA of the cells was stained with a mixture of ethidium bromide (5 $\mu$g/ml) and mithramycin (12·5 $\mu$g/ml) in Tris buffer.DNA distributions were recorded on a flow cytometer ICP 22 (Phywe, Göttingen, FRG) on line with a Wang computer 2200B (Wang, Tewksbury, U.S.A.). The fractions of cells in the various phases of the cell cycle were determined as described by Beck (1980). The absolute DNA content of the tumour cells was determined by calibrating the instrument with kidney cells, which are known to have the normal diploid DNA content (2c) of 6 pg/cell.

$^3$H-labelling indices were determined from tissue samples which had been incubated ***in vitro*** for 1 h at 37°C under high $O_2$ pressure with 10 $\mu$Ci/ml $^3$H-thymidine. ARGs of Feulgen-stained 3 $\mu$m slices were prepared using Ilford K2 emulsion and the gold-activation technique (Braunschweiger *et al.* (1976).

### *Histology*

Histological grading was performed according to the two systems introduced by Syrjänen & Hjelt (1978).

*Nuclear morphology.*—Grade I: spherical nuclei, delicate chromatin, inconspicuous nucleoli, rare mitotic figures. Grade II: spherical nuclei, distinct strands of chromatin, visible nucleoli, scattered mitotic figures. Grade III: anisonucleosis, coarse and clumped chromatin, prominent nucleoli, frequent mitotic figures. Grade IV: prominent anisonucleosis, many enlarged nuclei, coarse chromatin, prominent nucleoli, frequent mitotic figures.

*Combined histological grading* based on nuclear structure and the demarcation of the carcinoma from the surrounding renal tissue. Grade IA: well differentiated and demarcated. IB: well differentiated and poorly demarcated. IIA: moderately differentiated and well demarcated. IIB: moderately differentiated and poorly demarcated. IIIA: poorly differentiated and well demarcated. IIIB: poorly differentiated and poorly demarcated.

### RESULTS

Figure 1 shows FCM DNA histograms of normal kidney tissue and 2 renal adenocarcinomas. The kidney cells (A) were used as a standard for determining the DNA content of the tumour cell lines. B shows a purely diploid tumour, whereas the tumour of C contains a diploid (2c DNA content) as well as a hyperdiploid (3·5c) cell population.

Fig. 2 shows a tumour with pronounced differences between the periphery and the centre, and demonstrates the evaluation procedure for the FCM histograms. The raw data of the kidney tissue (A) and the tumour periphery (B) look rather similar because of the semi-logarithmic plot, but after background subtraction and correction for clumped cells the ($G_2+M$) peak of the tumour appears considerably higher than that of the normal kidney tissue. In

FIG. 1.—DNA histograms of kidney tissue (A), of a renal carcinoma containing only diploid tumour cells (B), and of a renal carcinoma containing hyperdiploid as well as diploid tumour cells (C).

this particular tumour a hyperdiploid cell population, in addition to the diploid one, was found in the sample from the centre (C). Since the renal carcinomas are usually large, the collected samples represent only a very small part of the tumour. If a small hyperdiploid population were confined to a distinct region, it could easily be missed by taking only 2 samples. However, for 18/21 tumours the aneuploid population was found both in the centre and at the periphery. In 3 cases, the hyperdiploid population appeared only in one of the 2 samples. This shows that the probability of missing the hyperdiploid population in *both* tumour samples is less than 1%.

Hyperdiploid cell populations were found in 21 tumours (*i.e.* 46%) of a total of 46. The results are compiled in Table I. For all diploid tumour-cell populations investigated, including those of Table I (population 1) an average of $5{\cdot}9 \pm 0{\cdot}5$ (s.e.) pg DNA per $G_1$ cell was obtained. This agrees well with the known value of 6 pg for human diploid cells. Therefore the ploidy of population 2 was calculated on the assumption that the accompanying population 1 had 2 complements of DNA. For all tumours with one cell population, the deviation from the mean was within statistical limits (according to a Dixon test at the 5% level) except for sample 10 in Table I. Therefore this tumour with 7·9 pg DNA content was assigned to be hyperdiploid and included in the table, despite having only 1 population. In 1 case (7) we measured a hypoploid population (1·5c) in addition to the diploid and the hyperdiploid ones. The percentages of cells in the 2 populations, listed in the last columns, differ considerably from sample to sample.

Table II shows the fractions of cells in the various phases of the cell cycle as determined by FCM. The mean values of S and $(G_2+M)$ fractions of tumours are clearly higher than those of normal kidney cells for unirradiated samples, due to the enhanced proliferation of the tumour cells. The S and $(G_2+M)$ fractions of irradiated tumours do not show any clear tendency, but the $(G_0+G_1)$ fractions appear to be higher than in the unirradiated tumours. In contrast, the results of the normal kidney cells are the same for irradiated and unirradiated samples. Since the tumours were randomly chosen for irradiation, this might indicate that irradiation reduced proliferation.

FIG. 2.—DNA histograms of normal kidney tissue (A), cells from the tumour periphery (B) and cells from the centre of the tumour (C), from sample 19 in Table I. The number of cells is plotted in log scale to identify small peaks. The solid line shows the computer fit of the peaks, the broken line the background subtraction, calculated mainly from the part of the histogram in front of the 1st peak. In the upper 2 charts the correction for clumped cells is demonstrated (·—·—). The contamination of the 2nd peak (4c) with clumped cells depends mainly on the number of cells in the 3rd peak (6c), which contains the clumps made up of three 2c cells.

Due to the great variation from sample to sample, no differences between means are statistically significant. The fractions of cells in the phases of the cell cycle also differed in some cases between periphery and centre of the tumour. Since the means ($\pm$s.e.) of the ($G_0+G_1$) fractions of all diploid tumours were $93{\cdot}3\pm0{\cdot}8\%$ for the periphery and $92{\cdot}6\pm1{\cdot}0\%$ for the centre, the data could be combined (Table II). On the histological slides, however, necrosis was more extended in the centre than near the tumour periphery. Generally, this does not influence the FCM results, since the necrotic cells are decomposed when preparing single-cell suspensions, so that only intact cells are recorded in the FCM. Our findings are in accordance with those of Rabes (1980), who, studying the proliferation in different intact tumour regions of renal carcinoma by ARG, found only small differences in labelling indices.

The labelling index was determined by ARG for 3 diploid tumours after incubating the samples with [$^3$H]dT *in vitro*. This was done to countercheck the FCM results, since it is rather difficult to determine S-phase fractions below 5% with adequate accuracy by FCM. The results obtained by the 2 methods agree within 1·5% (Table III), confirming the reliability of our FCM data.

The correlation between ploidy determined by FCM and histological grading is shown in Tables IV and V. Obviously, more hyperdiploid tumours are of nuclear Grades III and IV than are diploid tumours (Table IV), but there are also hyperdiploid tumours of Grades I and II and diploid tumours of Grade III. On the other hand, all Grade IV tumours and 5/7 of Grade III are hyperdiploid. That means the correlation is not bidirectional: all Grade IV tumours are hyperdiploid, but not all hyperdiploid tumours are Grade IV.

In Table V the correlation between the combined histological grading and FCM is shown. For the classification based on differentiation, denoted I–III, the results correspond to those presented in Table IV. Infiltrative growth, denoted B, was observed for all tumours of the most malignant Grade III, whether diploid or

TABLE I.—*DNA content, ploidy of population 2, and % cells in the 2 populations, in tumours with hyperdiploid cell populations. The results presented are from the tumour centre, except for sample* 11, *where population* 2 *appeared only in the periphery. In samples* 1 *and* 19 *the aneuploid population was measured only in the centre sample. In all other cases 2 populations were found in both centre and periphery. For the sake of clarity, only the data from the tumour centre are presented*

| Pre-irrad. dose (Gy) | Sample | Mean DNA content of population (pg/nucleus) 1 | 2 | Ploidy of population 2 | % of cells in population 1 | 2 |
|---|---|---|---|---|---|---|
| 0 | 1 | 6·5 | 13·4 | 4·1 | 67 | 33 |
| | 2 | 5·5 | 15·9 | 5·8 | 6 | 94 |
| | 3 | 6·0 | 10·6 | 3·5 | 56 | 44 |
| | 4 | 6·5 | 7·9 | 2·4 | 57 | 43 |
| | 5 | 5·3 | 8·8 | 3·3 | 62 | 38 |
| | 6 | 6·0 | 8·3 | 2·8 | 35 | 65 |
| | 7 | 6·0 | 4·6/9·4 | 1·5/3·1 | 3 | 85/12 |
| | 8 | 6·7 | 13·7 | 4·1 | 85 | 15 |
| | 9 | 6·2 | 10·8 | 3·5 | 74 | 26 |
| | 10 | — | 7·9 | 2·6 | — | 100 |
| | 11 | 6·2 | 11·3 | 3·6 | 91 | 9 |
| 25 | 12 | 5·8 | 11·1 | 3·8 | 92 | 8 |
| | 13 | 6·2 | 11·6 | 3·7 | 49 | 51 |
| | 14 | 5·8 | 11·1 | 3·8 | 54 | 46 |
| | 15 | 7·3 | 13·3 | 3·6 | 64 | 36 |
| | 16 | 6·0 | 8·4 | 2·8 | 75 | 25 |
| | 17 | 6·7 | 13·2 | 3·9 | 77 | 23 |
| 15 | 18 | 5·1 | 6·2 | 2·4 | 89 | 11 |
| | 19 | 6·7 | 11·3 | 3·4 | 74 | 26 |
| | 20 | 5·5 | 10·2 | 3·7 | 34 | 66 |
| | 21 | 6·2 | 9·0 | 2·9 | 57 | 43 |

TABLE II.—*Percentage of cells in the various phases of the cell cycle as determined by FCM. The means* ($\pm$ *s.e.*) *were calculated for samples taken from the periphery and the centre of the tumours*

| Sample | Pre-irrad. (Gy) | No. cases | % cells in phases $G_0+G_1$ | S | $G_2+M$ |
|---|---|---|---|---|---|
| Kidney tissue | 0 | 10 | 97·3±0·4 | 1·0±0·2 | 1·7±0·4 |
| | 25 | 10 | 96·9±0·4 | 1·1±0·2 | 2·0±0·5 |
| | 15 | 10 | 97·0±0·2 | 1·3±0·2 | 1·7±0·2 |
| Diploid kidney tumours | 0 | 9 | 91·7±1·4 | 4·2±0·9 | 4·1±0·9 |
| | 25 | 6 | 94·4±0·8 | 1·3±0·3 | 4·3±0·8 |
| | 15 | 10 | 94·1±0·8 | 1·8±0·4 | 4·1±0·5 |
| Hyperdiploid population of kidney tumours | 0 | 10 | 84·0±2·6 | 7·5±1·5 | 8·5±1·2 |
| | 25 | 4 | 88·3±3·1 | 7·3±3·3 | 4·4±1·1 |
| | 15 | 3 | 91·8±3·1 | 5·2±2·3 | 3·0±1·0 |

hyperdiploid. The tumours classified as Grades I and II according to differentiation were partly well demarcated (A), partly infiltrative (B), both in the diploid and hyperdiploid groups.

Table VI shows the clinical results in comparison to ploidy determined by FCM and to histological grading. During the follow-up for 6 month to 2 years, 9% of the patients with diploid tumours, but 47% with hyperdiploid tumours, died or developed multiple metastases, indicating that patients with aneuploid cell lines in the tumour have a worse prognosis than those with diploid tumours. The nuclear grading also correlates well with prognosis.

TABLE III.—*% cells in S, determined by ARG and FCM for 3 diploid tumours*

| Pre-irradiation dose (Gy) | Site of sampling | % cells in S, determined by ARG | FCM |
|---|---|---|---|
| 0 | Periphery | 3·3 | 2·0 |
| | Centre | 3·1 | 2·0 |
| 0 | Periphery | 7·9 | 7·0 |
| | Centre | 1·2 | 0 |
| 25 | Periphery | 2·5 | 1·0 |
| | Centre | 1·5 | 1·0 |

TABLE IV.—*Ploidy of human kidney tumours determined by FCM, compared to nuclear grading from histological slides*

| Histological grading | No. of tumours in group I | II | III | IV | Total | % tumours in groups III+IV |
|---|---|---|---|---|---|---|
| FCM | | | | | | |
| Diploid | 8 | 9 | 2 | 0 | 19 | 11 |
| Hyperdiploid | 2 | 7 | 5 | 6 | 20 | 55 |
| % Hyper-diploid | 20 | 44 | 71 | 100 | — | — |

### DISCUSSION

The ploidy of tumour cells is one of the characteristics easily accessible by flow cytometry. We found that 46% of the renal carcinomas investigated contained a hyperdiploid line. The ratio of hyperdiploid cells varies strongly from sample to sample (Table I). In principle, the diploid population in the FCM histograms might also include normal kidney cells and macrophages. Histological inspection, however, showed that the great majority of cells were tumour cells in all samples investigated. The tumours were classified as aneuploid only when a second peak in addition to the diploid one appeared in the FCM histogram or, as in one exception, when the difference from the DNA content of normal tissue was highly significant. However, a small hypodiploid or hyperdiploid population with DNA content near 2c cannot be separated from a greater 2c peak in the histogram. Considering the coefficients of variation of 4–9% in the histograms, we estimated that a near-diploid peak could only be identified if it had a DNA content at least 20% lower or higher than the corresponding 2c peak. That means that our "hyperdiploid" group contains only tumours with cell lines of DNA content 2·4c or more (*cf.* Table I, column 5). In some tumours, the cell population 2 with the higher DNA content has a ploidy close to

TABLE V.—*Ploidy of human kidney tumours determined by FCM compared to combined histological grading. Grades I–III reflect nuclear morphology, A and B denote demarcation and infiltration, respectively.*

| Histological grading | Number of tumours in group IA | IB | IIA | IIB | IIIA | IIIB | Total | % tumours in Group IIIB |
|---|---|---|---|---|---|---|---|---|
| FCM | | | | | | | | |
| Diploid | 6 | 3 | 4 | 5 | 0 | 1 | 19 | 5 |
| Hyperdiploid | 1 | 1 | 5 | 3 | 0 | 10 | 20 | 50 |
| % Hyperdiploid | 14 | 25 | 56 | 38 | — | 91 | | |

TABLE VI.—*Results of clinical treatment of patients with renal carcinomas in relation to ploidy of tumours determined by FCM, and to histological grading. The period of follow-up varied from 6 months to 2 years*

| Tumour classification: Method | Grade | No. Total | No. of patients with multiple metastases: dead | alive | % of all patients with multiple metastases |
|---|---|---|---|---|---|
| Flow cytometry | Diploid | 11 | 1 | 0 | 9 |
| | Hyperdiploid | 17 | 6 | 2 | 47 |
| Nuclear morphology | I | 6 | 0 | 0 | 0 |
| | II | 12 | 2 | 0 | 17 |
| | III | 5 | 2 | 0 | 40 |
| | IV | 5 | 3 | 2 | 100 |

4c; *i.e.* the peak appears at nearly the same position as the $(G_2+M)$ peak of population 1 with diploid DNA content. These samples were classified as hyperdiploid only if S-phase cells did not appear between the 2c and 4c peaks, and if, in addition, a peak at 8c $(G_2+M)$ cells of population 2 appeared, which was smaller than the 6c peak, so that it could not be caused by clumping of diploid cells.

The DNA content of a variety of human tumours, including 9 renal carcinomas, was measured by Atkin & Kay (1979). They found hyperdiploid tumour lines in 50–70% of 1465 cases, depending on the type of tumour. This is in accordance with our result of 46% hyperdiploid renal carcinomas. On the other hand, Barlogie *et al.* (1980) observed aneuploidy in 91% of several different types of solid human tumours, and concluded that the great majority of all solid tumours have hyperdiploid abnormalities. However, in their study the cells were classified as aneuploid when the DNA content exceeded 1·05 times normal. When the near-diploid cases (< 2·4c) are excluded, only 63% remain in the aneuploid group, which is compatible with our results.

The proliferative state of the tumours can be inferred from the fractions of cells in the various phases of the cell cycle. When the fractions of S and $(G_2+M)$ cells are low, as in renal carcinomas, the results obtained by FCM show rather large uncertainties (Baisch *et al.*, 1982), which are mainly because background subtraction has to be performed in evaluating FCM data of tumour samples (Beck, 1980). In contrast to FCM, even a few cells in S phase can be clearly identified by ARG. The good agreement between our ARG and FCM results (Table III) shows that the low fractions of cells in S, obtained by FCM, are quite reliable. Our ARG results agree also with the findings of Rabes (1980), who reported labelling indices for renal carcinomas of 1–5·8%. For the hyperdiploid populations we measured higher fractions in $S+(G_2+M)$ than in diploid populations (Table II). This finding is also supported by our observation that the tumours containing hyperdiploid lines show more mitoses than the diploid ones, and are hence more frequently classified as Grade III or IV by histology (see Table IV).

The pre-irradiated tumours showed little change in size of S and $(G_2+M)$ fractions from the unirradiated ones (Table II). From a cell-kinetic point of view one should expect that irradiation with 15 Gy the day before removal of the tumour should cause a measurable $G_2$ block, whereas 25 Gy 6 weeks before nephrectomy and FCM measurement should give the tumours time to recover and attain a balanced growth. But the results for the different treatment protocols were similar. The reason may be that any differences, if they exist, would be too small to be detected by FCM, since the proportion of hyperdiploid cells varies greatly from tumour to tumour, and the fraction of non-proliferating cells (as indicated by the large proportion in $G_0+G_1$) is high.

Aneuploidy measured by FCM correlates with histological grading: 55% of the hyperdiploid tumours are of nuclear Grade III and IV, compared with only 11% of the diploid tumours (Table IV). On the other hand, all tumours of Grade IV and 67% of Grade III were hyperdiploid. The hyperdiploid DNA content obviously does not always lead to morphological changes in the cell nucleus, but the increase in DNA content may be a prior event which eventually causes morphological changes. Some of the diploid tumours are also of high nuclear grade. Aneuploidy thus seems to be a possible but not necessary result of malignant transformation. For the characterization of a tumour, the ploidy level may be a useful additional parameter to the histological grading. The demarcation and infiltration (denoted A and B in the combined histological grading system) does not correlate to the ploidy for the lower Grades I and II, but all Grade III tumours were poorly demarcated (B). Except for the most malignant tumours, infiltration seems to be a characteristic uncorrelated to cell features.

The aneuploidy measured by FCM is shown to be relevant for prognosis. Nearly 50% of the patients with hyperdiploid tumours had a bad prognosis, whereas only 1 out of 11 patients with diploid tumours have died up to now. However, the period of follow-up was only 6 months to 2 years; *i.e.*, only the early multiple metastases and deaths were observed. The number of tumours in Table VI is less than the total number in the study, because in some cases the fate of the patients was uncertain. A slight correlation between aneuploidy and poor prognosis was also found by Atkin & Kay (1979) for various types of tumours. Our present data indicate that this tendency is more pronounced for renal carcinomas. We also found a good correlation between histological grading and prognosis (Table VI). This generally agrees with the follow-up study based on histological grading by Syrjänen & Hjelt (1978). They showed that patients with renal carcinomas of higher grades had considerably lower 5-year survivals than the low-grade groups.

In our study, all Grade IV tumours were hyperdiploid and had a bad prognosis (Table IV). On the other hand, the tumours of the lower grades were not always diploid. Even in the lowest Grade I group, 2/10 tumours were hyperdiploid. Obviously the ploidy characteristic is not strictly correlated with nuclear morphology. Interestingly, in the group of patients who died during the period of observation, both Grade II tumours and 1/2 Grade III tumours were hyperdiploid. The 2 patients with Grade II tumours died about 2 years after nephrectomy. For the remaining low-grade, hyperdiploid tumours the follow-up period has probably been too short. But the present results already indicate that ploidy can be used as an independent parameter with respect to prognosis. It is hoped that a grading system based on both FCM ploidy and histological grades might improve the prognostic possibilities for this particular human tumour.

This work was supported by Deutsche Forschungsgemeinschaft, Bonn. Radiotherapy with 25 Gy was performed by Professor H.-D. Franke, Department of Radiotherapy, University Hospital Hamburg. Radiotherapy with 15 Gy was performed by Dr M. L. Arnal, Department of Radiotherapy, General Hospital Altona. Nephrectomy of these latter tumors was performed by Professor J. Kaufmann, Department of Urology, General Hospital Altona. We thank Ms D. Lensch and Ms H. Bollmann for skilful technical assistance.

### REFERENCES

ATKIN, N. B. & KAY, R. (1979) Prognostic significance of modal DNA value and other factors in malignant tumours, based on 1465 cases. *Br. J. Cancer*, **40**, 210.

BAISCH, H., BECK, H.-P., CHRISTENSEN, I. J. & 10 others (1982) A comparison of mathematical methods for the analysis of DNA histograms obtained by flow cytometry. *Cell Tissue Kinet.*, in press.

BARLOGIE, B., GÖHDE, W., JOHNSTON, D. A. & 4 others (1978) Determination of ploidy and proliferative characteristics of human solid tumours by pulse cytophotometry. *Cancer Res.*, **38**, 3333.

BARLOGIE, B., SCHUMANN, J., GÖHDE, W. & 4 others (1980) Cellular DNA content as a marker of neoplasia in man. *Am. J. Med.*, **69**, 195.

BECK, H.-P. (1980) Evaluation of flow cytometric data of human tumours. *Cell Tissue Kinet.*, **13**, 173.

BICHEL, P., FREDERIKSEN, P., KJAER, T., THOMMESEN, P. & VINDELØV, L. L. (1977) Flow microfluorometry and transrectal fine-needle biopsy in the classification of human prostatic carcinoma. *Cancer*, **40**, 1206.

BRAUNSCHWEIGER, P. G., POULAKOS, L. & SCHIFFER, L. M. (1976) *In vitro* labeling and gold activation autoradiography for determination of labeling index and DNA synthesis in tissues of solid tumors. *Cancer Res.*, **36**, 1748.

MØRK, S. J. & LAERUM, O. D. (1980) Modal DNA content of human intracranial neoplasms studied by flow cytometry. *J. Neurosurg.*, **53**, 198.

RABES, H. M. (1980) Growth kinetics of human renal adenocarcinoma, *UICC Tech. Rep. Series*, **49**, 78.

ROTERS, M., LINDEN, W. A. & HEIENBROK, W. (1978) Comparison of three different methods for the preparation of human tumours for flow cytometry (FCM). In *Third Int. Symp. Pulse Cytometry*, (Ed. Lutz) Ghent: European Press. p. 423.

SCARFFE, J. H., HANN, I. M., EVANS, D. I. K. & 4 others (1980) Relationship between pretreatment proliferative activity of marrow blast cells and prognosis of acute lymphoblastic leukaemia of childhood. *Br. J. Cancer*, **41**, 764.

SYRJÄNEN, K. & HJELT, L. (1978) Grading of human renal adenocarcinoma. *Scand. J. Urol. Nephrol.*, **12**, 49.

TRIBUKAIT, B. & EPOSTI, P. (1976) Comparative cytofluorometric and cytomorphologic studies in non-neoplastic and neoplastic human urothelium. (Eds. Göhde *et al.*). In *Pulse-Cytophotometry*, Ghent: European Press. p. 176.

VAN DER WERF-MESSING, B., VAN DER HEUL, R. O. & LEDEBOER, R. C. (1978) Renal cell carcinoma trial. *Cancer Clin. Trials*, p. 13.